# *Vibrio parahaemolyticus*, Southern Coastal Region of China, 2007–2012

**DOI:** 10.3201/eid2004.130744

**Published:** 2014-04

**Authors:** Yinghui Li, Xu Xie, Xiaolu Shi, Yiman Lin, YaqunQiu, Jin Mou, Qiongcheng Chen, Yan Lu, Li Zhou, Min Jiang, Honghu Sun, Hanwu Ma, Jinquan Cheng, Qinghua Hu

**Affiliations:** Shenzhen Major Infectious Disease Control Key Laboratory, Shenzhen Center for Disease Control and Prevention, Shenzhen, China (Y. Li, X. Xie, X. Shi, Y. Lin, Y. Qiu, J. Mou, Q. Chen, Y. Lu, L. Zhou, M. Jiang, H. Ma, J. Cheng, Q. Hu);; Sichuan University, Chengdu, China (H. Sun);; Shenzhen University, Shenzhen (Q. Hu)

**Keywords:** Vibrio parahaemolyticus, infectious diarrhea, epidemic, China, bacteria, Vibrio

## Abstract

We analyzed the prevalence and characteristics of *Vibrio parahaemolyticus* among patients with acute infectious diarrhea in the southern coastal region of China. *V. parahaemolyticus* was the leading cause of bacterial infectious diarrhea in this region during 2007–2012. Serotype O3:K6 strains were most common, followed by serotypes O4:K8 and O3:K29.

*Vibrio parahaemolyticus*, a halophilic bacterium, is recognized as a major cause of acute gastroenteritis worldwide, often associated with the consumption of raw or undercooked shellfish. *V. parahaemolyticus* infections are caused by diverse serotypes; however, serotype O3:K6 has been reported to be dominant and has been a widespread serotype since 1997 (*1*).

*V. parahaemolyticus* has been the leading cause of foodborne outbreaks and bacterial infectious diarrhea in China since the 1990s, especially in coastal regions (*2*,*3*). Serotype O3:K6 was documented as the dominant serotype in Zhejiang Province, China, in 2002 and was proven to be a pandemic clone in 2008 (*4*). However, long-term fluctuation in the frequency of infections with the pandemic strains of *V. parahaemolyticus* remains unknown.

In 2007, laboratory-based surveillance for acute infectious diarrhea at 11 sentinel hospitals was established in Shenzhen City in the southern coastal region of China with *V. parahaemolyticus* as one of the target pathogens. To characterize *V. parahaemolyticus* infections and clarify its prevalence in this region, we analyzed all *V. parahaemolyticus* cases captured by this surveillance during 2007–2012.

## The Study

Surveillance was conducted among outpatients who had >3 loose or liquid stools during a 24-hour period but lasting <14 days. A total of 1,488 *V. parahaemolyticus* infections were identified from 24,696 enrolled outpatients (6.0% of outpatients). More than half of the patients (835; 56.1%) were male. Patients ranged in age from 4 months to 84 years (median 27 years); 1,383 (92.9%) patients were 15–39 years of age. Most (914; 61.4%) patients were part of the large transient population living in Shenzhen. Of all patients with *V. parahemoliticus* infection, 1,150 (77.3%) had watery diarrhea, 1,176 (79.0%) had abdominal pain, 730 (49.1%) had vomiting, 206 (13.8%) had fever, and 4 (0.3%) had blood in stools.

Obvious monthly peaks of *V. parahaemolyticus* infections were found during the warmer months (June–October). Up to 30% of diarrhea cases covered by the surveillance could be attributed to *V. paraheamolyticus* infections during this period.

All 1,488 *V. parahaemolyticus*isolates were serotyped by slide agglutination by using a commercial serum (Denka-Seiken Ltd., Tokyo, Japan); 47 serotypes were detected. The O3:K6 serotype was dominant throughout the surveillance years (996 isolates; 66.9%), followed by O4:K8 (156 isolates; 10.5%) and O3:K29 (51 isolates; 3.4%). However, O3:K29 appeared more frequently during 2009 and 2010. Four other serotypes occurred as clusters in different years: O1:KUT in 2008, O1:K56 in 2010, O4:K68 in 2010, and O5:K68 in 2009 (Table 1).

**Table 1:** Serotype composition of 1,488 *Vibrio parahaemolyticus* isolates from patients with acute diarrhea, southern coastal region of China, 2007–2012

Serotype	No. isolates						
	2007	2008	2009	2010	2011	2012	Total
O3:K6	153	94	180	210	198	161	996
O4:K8	12	10	49	42	25	18	156
O3:K29	2	1	24	18	5	1	51
O1:KUT	1	20	4	4	10	8	47
O1:K56	0	0	0	28	0	1	29
O1:K36	5	4	3	9	2	1	24
O4:K9	1	5	6	1	3	7	23
O4:K68	2	4	2	10	2	0	20
O5:K68	2	0	10	3	1	0	16
O1:K25	5	1	3	3	2	1	15
O11:K36	0	0	5	7	1	1	14
O2:K3	0	3	3	3	0	4	13
O3:KUT	0	4	3	1	0	1	9
O4:K11	2	0	2	1	0	1	6
O4:K55	0	1	0	2	2	1	6
O1:K41	1	0	1	0	1	2	5
O4:K13	0	1	4	0	0	0	5
O4:KUT	0	1	1	0	0	3	5
O8:K41	1	1	0	0	0	3	5
OUT:KUT	1	1	1	0	0	1	4
O6:K18	0	1	1	0	0	1	3
O8:K21	0	0	2	0	0	1	3
OUT:K68	3	0	0	0	0	0	3
O1:K1	1	1	0	0	0	0	2
O1:K68	0	0	0	2	0	0	2
O1:K69	0	0	0	0	2	0	2
O10:K52	0	0	0	0	0	2	2
O3:K59	0	0	0	1	0	1	2
O4:K12	0	0	0	0	0	2	2
O1:K5	0	0	0	0	0	1	1
O10:KUT	0	0	0	0	1	0	1
O11:K19	1	0	0	0	0	0	1
O11:KUT	0	0	0	0	1	0	1
O2:KUT	0	0	0	1	0	0	1
O3:K1	0	0	1	0	0	0	1
O3:K2	0	0	0	1	0	0	1
O3:K28	0	0	0	1	0	0	1
O3:K41	0	0	0	1	0	0	1
O3:K45	0	1	0	0	0	0	1
O3:K54	1	0	0	0	0	0	1
O3:K56	0	0	1	0	0	0	1
O3:K7	0	0	0	0	0	1	1
O4:K63	0	0	0	1	0	0	1
O8:KUT	0	0	0	0	0	1	1
OUT:K19	1	0	0	0	0	0	1
OUT:K44	0	1	0	0	0	0	1
OUT:K55	0	0	0	0	1	0	1

Representative strains were selected for PCR of the virulence genes thermostable direct hemolysin (*tdh*) and TDH-related hemolysin (*trh*) (*5*,*6*). A total of 833 isolates covering all 47 serotypes (1–560 isolates for each serotype) were screened. Most (788; 94.6%) strains were *tdh^+^trh^–^*; 28 isolates representing 5 serotypes were *tdh^+^trh^+^* (O1:KUT, n = 18; O4:K12, n = 3; O1:K69, n = 4; O10:K52, n = 2; O4:K55, n = 1), and 17 isolates of 10 serotypes were *tdh^–^trh^–^* (O1:KUT, n = 5; O3:K6, n = 4; O1:K1, O1:KUT, O3:K1, O3:KUT, O4:K63, O10:KUT, OUT:K19, and OUT:K55, n = 1 each).

A total of 196 *tdh^+^trh^–^* isolates, representing the leading 10 serotypes (6–75 isolates for each serotype), were selected for group-specific PCR (GS-PCR) of *toxRS*/new sequence (*7*). Results demonstrated that pandemic genotype strains (110; 56.12%) prevailed among the leading 10 serotypes in Shenzhen. Most (68; 90.7%) O3:K6 isolates; all O1:K36, O4:K68, O5:K68, and O1:K25 isolates; and 6 (28.6%) O1:KUT isolates gave positive results by GS-PCR, whereas results were negative for the other 4 serotypes.

We analyzed 127 isolates by using the *V. parahaemolyticus*multilocus sequence typing (MLST) scheme (http://pubmlst.org/vparahaemolyticus) (Figure). Clonal complex 3 (CC3) predominated (93 isolates; 73.2%). A new clonal complex, CC120, and sequence type (ST), 265, a presumed new ancestor of CC345, were identified (Table 2).

**Figure:**
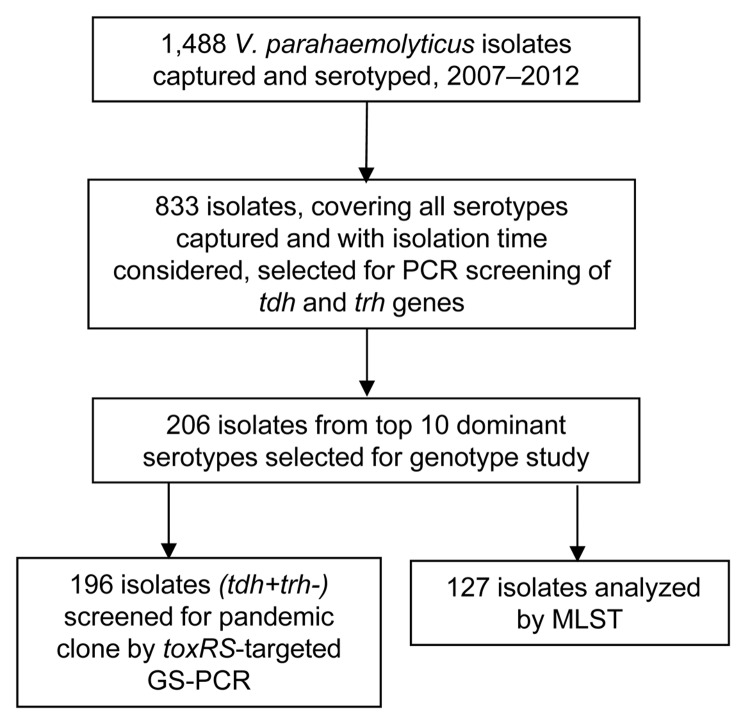
Study design and eligibility for serotype and genotype analysis of *Vibrio parahaemolyticus* isolates from patients with acute diarrhea, southern coastal region of China, 2007–2012.

**Table 2:** Results of GS-PCR and MLST analysis of the 10 mostly commonly found serotypes of *Vibrio parahaemolyticus* isolates from patients with acute diarrhea, southern coastal region of China, 2007–2012*

Serotype	GS-PCR, n = 196			MLST, n = 127		
	No. positive, n = 110	No. negative, n = 86		No. isolates tested	ST (no. isolates)	CC
O3:K6	68	7		64	ST3 (60), ST487 (1), ST489 (1), ST526 (1)	CC3
					ST497 (1)	Singleton
O4:K8	0	30		16	ST265 (14), ST189 (1), ST438 (1)	CC345
O3:K29	0	14		13	ST120 (11), ST480 (2)	CC120
O1:KUT	6	15		3	ST3 (3)	CC3
O1:K56	0	10		8	ST8 (8)	CC8
O1:K36	9	0		7	ST3 (7)	CC3
O4:K9	0	10		3	ST332 (3)	Singleton
O4:K68	10	0		5	ST3 (5)	CC3
O5:K68	6	0		3	ST3 (3)	CC3
O1:K25	11	0		5	ST3 (4)	CC3
					ST481 (1)	Singleton

*GS-PCR, group-specific PCR; MLST, multilocus sequence typing; ST, sequence type; CC, clonal complex.

## Conclusions

During 2007–2012, *V. parahaemolyticus* was the dominant bacterial cause of acute diarrhea in the southern coastal region of China, surpassing *Salmonella* spp., diarrheagenic *Escherichia coli*, and *Shigella* spp. (data not shown). These findings differ from those for central and northern regions of China (*8*,*9*). Most case-patients in this surveillance were 15–39 years of age. The distribution of the 47 serotypes detected revealed the diversity of *V. parahaemolyticus*, which might explain the continuing epidemic of *V. parahaemolyticus* infections in this region.

*V. parahaemolyticus* serotype O3:K6, which emerged worldwide in 1997 as a pandemic clone and spread throughout Asia and to the Americas, Europe, and Africa (*1*), has been dominant in Shenzhen Province. Most (68; 90.7%) O3:K6 isolates tested were new clones, defined by *toxRS*-targeted GS-PCR, providing evidence that pandemic O3:K6 has spread to China. Most (63; 98.5%) serotype O3:K6 strains were identified as CC3 by MLST; serotype O1:KUT, O1:K36, O4:K68, O5:K68, and O1:K25 strains were positive by GS-PCR in our study and mostly documented as O3:K6 serovariants (*1*). These results indicate the long-term evolution of O3:K6 in Asia.

*V. parahaemolyticus* serotype O4:K8 has been an epidemic strain in Asia (*10*,*11*) and reported in Peru (*12*) but has been rarely seen in North America, Africa, and Europe. Serotype O4:K8 isolates from this study expressed a nonpandemic genotype. We presume that the evolution of O4:K8 was affected by local mutation and recombination rather than by a global pandemic, similar to a finding reported in Japan in 2007 (*13*). Notably, ST265 was predominant among strains with serotype O4:K8, whereas ST345, the previously considered founder of CC345, was not found (Table 2). Although ST265 might be a potential branch from ST345 in other regions, our findings strongly suggest that ST265 should be considered the epidemic clonal founder of CC345 in China. Overall, serotypes O3:K6 and O4:K8, stable subpopulations of the diverse *V. parahaemolyticus* population in our surveillance, have clearly been epidemic in China.

*V. parahaemolyticus* serotypes O3:K29 and O1:K56 were mainly reported in Asia, with nonpandemic groups identified in Japan (*10*,*14*). Our study showed that prevalence of serotype O3:K29 *V. parahaemolyticus* suddenly fluctuated during 2009 and 2010 and prevalence of O1:K56 fluctuated in 2010, but no focal outbreaks were confirmed; this finding indicates that sporadic outbreaks might have occurred. In addition, serotype O3:K29 isolates were identified as ST120 and the newly determined ST480, both belonging to CC120. Limited information could be obtained from the MLST database about the O1:K56 strains, and the isolates we tested were classified as ST8.

Further, our study found *V. parahaemolyticus* serotype O1:KUT might contain >1 character K antigens; however, 18 isolates harbored both *tdh* and *trh* genes, a combination that is not found frequently. Therefore, an O1:KUT epidemic clone might be prevalent in this region.

Whereas *V. parahaemolyticus* often is associated with the consumption of raw or undercooked shellfish, data from this surveillance program showed that most patients were transient residents who lived in rural areas and seldom ate seafood. However, epidemiologic data showed that *V. parahaemolyticus* infection was associated with eating outdoors and consumption of salad vegetables. Cross-contamination in food processing might be the source of infection; further epidemiologic investigation is under way.

In summary*, V. parahaemolyticus* has been prevalent for a long time in the southern coastal region of China, and diverse serotypes and multiple clones of the bacterium are circulating. On the basis of successful efforts to reduce prevalence of *V. parahaemolyticus* infections in Japan (*15*), we suggest holistic approaches involving regulations and guidance on fishery products and food hygiene to decrease the incidence of these infections in China.
